# P-504. Eight Years of Maternal Syphilis in Northern Mexico: Clinical and Demographic Profiles and Congenital Outcomes

**DOI:** 10.1093/ofid/ofaf695.719

**Published:** 2026-01-11

**Authors:** Abril M Gutiérrez-Castro, Paola Quintanilla-Urdiales, Rocio Ximena Sandoval-Orozco, Rubén G Valadez-Mata, Ian Carlo Pineda-Fierro, Judith Estela Guzman Garcia, Jessica Guerra-Díaz, Oscar Tamez-Rivera, Lindsay Ariadna Concha-Mora

**Affiliations:** Pediatric Residency Program, Programa Multicéntrico de Especialidades Médicas ITESM- SSNL, Tecnológico de Monterrey. Escuela de Medicina y Ciencias de la Salud. Monterrey, México, Monterrey, Nuevo Leon, Mexico; Pediatric Residency Program, Programa Multicéntrico de Especialidades Médicas ITESM- SSNL, Tecnológico de Monterrey. Escuela de Medicina y Ciencias de la Salud. Monterrey, México, Monterrey, Nuevo Leon, Mexico; Tecnológico de Monterrey Campus Monterrey, Chihuahua, Chihuahua, Mexico; Hospital Universitario Dr. Jose Eleuterio Gonzalez, Guadalupe, Nuevo Leon, Mexico; Hospital Universitario Dr. Jose Eleuterio Gonzalez, Guadalupe, Nuevo Leon, Mexico; Tecnologico de Monterrey, Monterrey, Nuevo Leon, Mexico; Hospital Universitario Dr. José Eleuterio González, Monterrey, Nuevo Leon, Mexico; Tecnologico de Monterrey, Escuela de Medicina y Ciencias de la Salud, Monterrey, Nuevo Leon, Mexico; The Hospital for Sick Children, Toronto, ON, Canada

## Abstract

**Background:**

Maternal and congenital syphilis remain significant global health issues, with >500,000 cases annually worldwide and serious outcomes including stillbirth, prematurity, low birth weight, and neurological damage. In Mexico, the steady rise in maternal syphilis reflects persistent challenges in early detection and follow-up, particularly in primary care. While national surveillance systems capture cases of acquired and congenital syphilis, they often lack clinical detail on neonatal outcomes, limiting a comprehensive evaluation.

**Figure:**
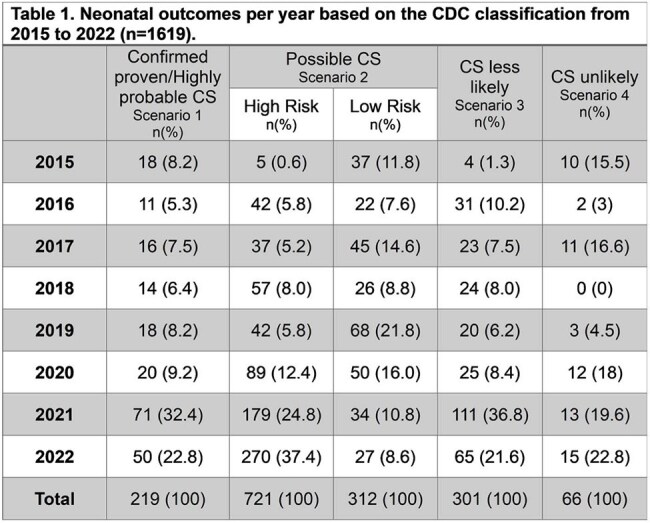

**Methods:**

We conducted a retrospective analysis of clinical data from pregnant women with a positive VDRL test and their newborns diagnosed at the Maternal and Pediatric Reference Hospital (HRMI) in NL, Mexico, between 2015 - 2022. Variables included maternal age, prenatal care, syphilis treatment, HIV coinfection, and neonatal outcomes classified according to CDC criteria. Descriptive statistics (frequencies, percentages, means, and ranges) were used to characterize maternal profiles and the congenital syphilis outcomes.

**Results:**

Clinical data from 1615 VDRL-positive pregnant women at the time of delivery along with their newborns (1619) were included. Mean maternal age was 22 ± 5.8 years. Alarmingly, only 923 (57%) attended adequate prenatal care, and only 426 (26%) received outpatient antibiotic treatment after the first positive VDRL test. Maternal HIV testing at the time of delivery revealed 22 women with a positive test (1.4%), all of which were novel diagnoses. Neonatal outcomes based on the CDC classification were as follows: 219 confirmed cases (13.5%), 1033 probable cases (63.8%), 301 possible cases (18.6%), and 66 syphilis exposure cases (4.1%). Of the probable cases, 721 newborns were considered at high-risk of developing congenital syphilis (44.5%) and were treated as such. A total of 57 stillbirths were documented (3.5%).

**Conclusion:**

Despite being a preventable condition, congenital syphilis (CS) remains a major burden in Northern Mexico. Low prenatal care coverage and missed treatment opportunities continue to drive high rates of adverse neonatal outcomes. Strengthening early detection and timely treatment during pregnancy is critical to prevent avoidable morbidity and mortality.

**Disclosures:**

All Authors: No reported disclosures

**Figure:** CS= Congenital Syphilis

